# Developmental Changes in the Relationship Between Character Reading Ability and Orthographic Awareness in Chinese

**DOI:** 10.3389/fpsyg.2019.02397

**Published:** 2019-10-25

**Authors:** Dan Lin, Jianhong Mo, Yingyi Liu, Hong Li

**Affiliations:** ^1^Department of Psychology, The Education University of Hong Kong, Tai Po, Hong Kong; ^2^Beijing Key Laboratory of Applied Experimental Psychology, National Demonstration Center for Experimental Psychology Education (Beijing Normal University), Faculty of Psychology, Beijing Advanced Innovation Center for Future Education, Beijing Normal University, Beijing, China

**Keywords:** orthographic, reading, longitudinal, Chinese, developmental stage

## Abstract

The present study examined developmental changes, over a 6-year period, in the relationship between character reading ability and orthographic awareness in Chinese from the first year of kindergarten to the third year of primary school in two separate samples: the kindergarten sample of 96 children was assessed three times in the first, second, and third years of kindergarten (K1, K2, K3) with 12-month intervals. The primary school sample of 204 children was assessed four times in the first and second semesters of grade 1 (P1-S1; P1-S2), first semester of grade 2 (P2-S1) and grade 3 (P3-S1), with the first three waves at 6-month intervals and the final wave at 12-month interval. Cross-lagged path analysis showed three developmental stages of the relationship between Chinese character reading and orthographic awareness. At stage 1, reading ability in K1 and K2 predicted subsequent orthographic awareness in K2 and K3. At stage 2, there was a bidirectional relationship between character reading and orthographic awareness from P1-S1 to P1-S2. At stage 3, orthographic awareness at P1-S2 and P2-S1 predicted subsequent character reading ability at P2-S1 and P3-S1, but the prediction from reading to orthographic awareness vanished at this stage. The results depict a full developmental picture of the changed relationship between Chinese character reading and orthographic awareness over time. Beginning readers demonstrated impressive abilities in discovering or extracting orthographic regularities with increased reading ability.

## Introduction

Reading is a developmental continuum rather than an all-or-none phenomenon (Teale and Sulzby, 1986). Emergent literacy skills of preliterate children are developmental precursors of conventional reading and writing, such as orthographic awareness. In a broad sense, orthographic awareness refers to the understanding of the print conventions or knowledge of word spelling (Conrad et al., 2013). Castles and Nation (2006) defined orthographic awareness as the sensitivity to orthographic regularities/rules in the script, which constrains the arrangement of the ordering of the internal structures. Studies in alphabetic languages have indicated that orthographic awareness at early stages is a strong predictor of subsequent reading acquisition (Badian, 2001; Boets et al., 2008). In recent decades, an increasing number of studies have identified the feature of orthographic awareness in Chinese, and its influence on Chinese word reading in typically developing children (e.g., Siok and Fletcher, 2001; Li et al., 2002; Ho et al., 2003b, 2012; Cheung et al., 2007; Tong et al., 2009; Tong and McBride-Chang, 2010), and those with dyslexia (e.g., Ho et al., 2002, 2004; Chung et al., 2010). However, little attention has been paid to examine the influence from word reading to orthographic awareness, or their bidirectional relationship. In two separate samples of kindergarten children (i.e., K1, K2, K3) and primary school students (i.e., P1, P2, P3), the present study aimed to capture the developmental change in the bidirectional relationship between Chinese character reading and orthographic awareness using the cross-lagged analysis approach.

### Features of Chinese Writing System

Chinese is typically described as a logographic script without grapheme–phoneme correspondences. In contrast to the linearity of an alphabetic language system, Chinese characters are visually compact, with a two-dimensional square-shaped configuration. Left–right (as in “
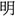
”), top-down (as in “
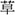
”), half-circle (as in “
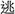
”), and a full circle (as in “
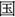
”) are four basic structures of character configurations. Strokes, radicals, and characters are the three different levels that constitute the Chinese writing system (Ding et al., 2004; Ho et al., 2012). Strokes are the basic structure unit of characters (e.g., “
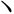
”
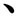
“
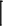
”
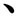
“
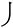
”). Unlike single letters in English, strokes do not carry any sound or meaning. Radicals are formed by interwoven strokes in patterns, which are the basic functional units of characters (e.g., “
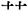
”
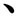
“
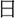
”
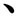
“
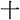
”). There are two types of radicals, semantic and phonetic, which convey semantic information or phonological information about the relevant characters to some extent. Radicals are the distinctive component in the Chinese writing system that has no parallel in alphabetic orthographies. Researchers have reported that over 80% of Chinese characters are compounds, consisting of semantic and phonetic radicals (Ho et al., 2003a), and that the number of semantic and phonetic radicals in Chinese is approximately 200 and 800, respectively. Thus, the mastery of radical related skills/knowledge is crucial to reading and spelling in Chinese (Ho et al., 2003a; Yeung et al., 2011).

Two issues related to radicals should be noticed. First, they can only “cue” the meaning or sound of a character; the information provided by radicals is not always complete or reliable. Semantic radicals provide approximate information about a character’s meaning or only indicate the semantic category of the characters they belong to Wei et al. (2014). To illustrate, characters containing “
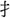
” are usually movements done by hand, such as “
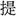
” [lift] and “
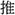
” [push]. As for the phonetic radicals, only about 40% of the compound characters share the exact same syllable with their phonetic radicals (e.g., Shu et al., 2003). The percentage drops to 25% when lexical tone is taken into consideration (Shu et al., 2003).

Second, there are positional constraints for radicals; the majority has fixed positions across characters. For illustration, ‘
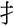
’ would only appear on the left side of a character, not on the right side. Generally speaking, semantic radicals are more likely to occur on the left or top side of the compound character, and phonetic radicals tend to appear on the right or bottom side (Ho et al., 2003a). Shu and Anderson (1997) reported that children from mainland China become aware of radicals as early as primary grade one, with explicit teaching by parents and teachers, and that radical awareness continues to develop even until fifth grade. Ho et al. (2003a) reported a similar developmental trend in Hong Kong children from the first to fifth grades, given the complexity of orthographic rules in Chinese, particularly different degrees of positional, semantic, and phonological regularities for radicals. Parents and teachers usually ask children to decode a novel compounding character into radicals, to identify and memorize the phonetic radical and semantic radical within a character, and to learn a group of characters sharing the same radical (e.g., Lin et al., 2012). Therefore, when Chinese children are learning to read, their sensitivity and knowledge of the orthographic structure, position, and function of radicals will increase greatly (Yeung et al., 2011).

### The Development of Orthographic Awareness

Given the Chinese language features discussed above, orthographic awareness in Chinese is defined as a child’s understanding of the orthographic rules for Chinese characters (Wei et al., 2014), which includes the knowledge of their legal components (stroke, radical, and whole character), and their positional and functional constraints. Ho et al. (2003b) proposed a developmental model of Chinese orthographic awareness, in which children acquire orthographic awareness with the sequence of character configuration knowledge, structural knowledge, radical information knowledge, positional knowledge, functional knowledge, amalgamation, and complete orthographic knowledge. The awareness of the internal structure of a character appears first, followed by the understanding of the compositional structure of a compound character. Next, the development of radical information and positional knowledge occurs almost simultaneously. Then the functional knowledge (which means that children understand the function that radicals convey in characters) occurs, followed by the amalgamation stage to integrate different types of knowledge.

Evidence from empirical studies (Ho et al., 2003a; Qian et al., 2015) has suggested that children showed the ability to differentiate the legal forms of characters from other visual symbols as early as the age of three. According to Ho et al. (2003a), the awareness of the compositional structure of compound characters in terms of radicals develops around first grade, but the children in their study did not understand the function of semantic radicals until P3. Partly consistent with Ho’s study, a recent study with a Beijing preschooler sample investigating the acquisition of orthographic awareness in Chinese (Qian et al., 2015) demonstrated that children could discriminate incorrect radical forms (within a character) with an accuracy of 87% at the age of five, but their understanding of positional and functional knowledge radicals remained rudimentary. While most studies suggested radical knowledge develops during the primary school period (Ho et al., 2003a; Tong and McBride-Chang, 2010; Luo et al., 2011; Wei et al., 2014), Chan and Nunes (1998) reported that Hong Kong kindergartners already demonstrated the awareness of radical knowledge to some extent. Children at 6 years old were able to reject the non-words and accept the pseudo-words, given the illegal position of radicals, and they were able to use semantic radicals to represent meaning in the creative spelling task; however, a more systematic use of phonological components as a clue to pronunciation was observed only in 9-year-olds.

To sum up, orthographic awareness emerges very early in a child’s development; however, it takes years for the sophisticated kinds of orthographic skills to develop fully, with increasing age and experience in Chinese learning. Given this developmental window, an investigation from the beginning of the kindergarten year through primary school grades is critical in obtaining a more systematic picture of the interplay of reading and orthographic knowledge. We anticipated that orthographic knowledge is crucial to successful character reading given the visual-orthographic complexity in Chinese. The influence from character reading to orthographic awareness is also anticipated.

### The Association Between Orthographic Awareness and Reading in Alphabetic Language and Chinese

Plenty of studies have examined the relationship between orthographic skills (the ability to form, store and access word representations) and reading ability in English (e.g., Cassar and Treiman, 1997; Burt, 2006; Castles and Nation, 2006). The role of orthographic skill in predicting word reading has been highlighted, given the inconsistency of grapheme–phoneme correspondences of English orthography (Treiman et al., 1995). For example, Cunningham et al. (2001) reported that orthographic knowledge at grade 2 predicted word reading accuracy at grade 3, even with phonological coding at grade 2 controlled. Recently the opposite prediction was evident in two longitudinal studies. Conrad and Deacon (2016) examined the bidirectional relationship between orthographic knowledge and word reading in a group of English-speaking children from grades 2 to 3. They found that orthographic knowledge was predicted by three types of reading skills (reading accuracy, reading efficiency and phonological decoding), but orthographic knowledge did not predict gains in word reading skills. Another longitudinal study (Burt, 2006) tracking children from grades 1 to 3 reported similar findings, that word reading predicted orthographic processing skill, regardless of grade level. In contrast, orthographic skills did not predict word reading. These studies in English suggested that the development of reading played an importantly role in fostering orthographic skills, compensating the long-standing traditional assumption that orthographic knowledge predicts the development of word reading.

As mentioned above, in recent decades, more and more research has addressed the contributions of orthographic knowledge to word reading in Chinese in typically developing children (e.g., Siok and Fletcher, 2001; Li et al., 2002; Ho et al., 2003b, 2012; Cheung et al., 2007; Tong et al., 2009; Tong and McBride-Chang, 2010), and research on Chinese children with dyslexia (e.g., Ho et al., 2002, 2004; Chung et al., 2010). Empirical evidence from intervention studies has also supported the contributions from orthographic knowledge to reading performance in preschoolers (Wang and McBride, 2016). These studies have shown consistently that orthographic awareness is important for Chinese reading development.

Tong et al. (2009) conducted a longitudinal study of K3 children in Hong Kong and found that orthographic awareness skills had consistently made a significant contribution to Chinese character reading, with age and vocabulary knowledge controlled. Similar results were found in a study of Chinese children from Mainland China (Wei et al., 2014). In a cross-sectional study, the authors compared the developmental changes from preschool to grade 3. Orthographic awareness, phonological awareness, and morphological awareness were identified to be the dominant predictor of reading in the first stage (preschool to grade 1), second (grade 2) and third stage (grade 3), respectively. Particularly, orthographic awareness measured via print knowledge and lexical decision task, exerted a significant role in word reading, after controlling for morphological awareness, phonological awareness, rapid automatized naming (RAN), and phonological memory from preschool to grade 1, but became insignificant in grades 2 and 3. Inconsistent results were reported by another study (Li et al., 2012), in which orthographic skills were found to be associated strongly with single-character reading in primary school (from grades 1 to 3) but not kindergarten (K2–K3), after controlling for phonological awareness, morphological awareness, RAN and visual skills. The inconsistent findings may be partly due to the test of different constructs in the studies. Orthographic awareness in Tong et al. (2009) and Wei et al. (2014) was basically tested on the configuration and structure knowledge, while Li et al. (2012) reported a higher level of orthographic conventions, such as radical awareness and positional rules. As reviewed above about the development of orthographic awareness, Chinese children in the preschool ages are mostly developing the positional knowledge but have limited access to functional knowledge, while in primary school ages, functional knowledge are progressively developing. Thus, it seems more comprehensive to have both positional and functional knowledge assessed for orthographic awareness in older children. In addition, the influence of reading to orthographic awareness was not tested or informed from these studies.

As the basic functional unit for word processing, radicals exhibit orthographic, phonological, and semantic properties; some studies have a particular focus on the awareness of radical predicting word reading development concurrently and longitudinally in Chinese children. Shu and Anderson (1999) reported that first and second graders could detect the non-characters obeying the positional convention of radicals, while third graders were able to make use of the semantic radical to derive the meaning of novel character countered, indicating the acquirement of radical knowledge. Consistently, Cheung et al. (2007) identified that successful access of semantic and phonetic radical knowledge concurrently predicted word reading in grade 4. Children’s understanding of semantic radical even contributed to their reading comprehension performance. In addition, three more recent studies that traced children from preschool to primary school provided a more comprehensive developmental picture of the association between orthographic awareness and reading ability. First, Tong and McBride (2014) asked Hong Kong Chinese children across different levels (kindergarten, 2^nd^, 5^th^ grades) to make up a novel character that looked most like a real character with two given radicals. The results showed that kindergartners showed some awareness of positional constraints and were able to produce orthographically legal pseudo-characters with an accuracy rate at approximately 40–60%. The performance increased with grades. Tong et al. (2017) compared the development of positional, phonetic, and semantic cues of radicals and their roles in reading development, suggesting a transition from position-based to a meaning-based focus in learning Chinese from K3, P2 to P5. With the age increasing, the role of semantic radical awareness increased while positional regularity awareness decreased in Chinese reading. Yeung et al. (2011) found that semantic radical knowledge (measured by the pseudo-character meaning judgment task) is a significant predictor of Chinese word reading in primary school grade 1 students, even after controlling for other meta-linguistic skills.

To summarize, these findings support the notion that orthographic awareness, in terms of sensitivity to the configuration, structural knowledge, positional and functional radical awareness, is a strong contributor to word reading, independent from other meta-linguistic skills such as phonological and morphological awareness. Despite the general contention that print exposure and reading experiences will stimulate the development of orthographic awareness, there is a lack of empirical studies directly testing the prediction of reading on the development of orthographic knowledge. Therefore, a logical step is to investigate the bidirectional association between orthographic awareness reading ability.

### The Present Study

To depict the development picture of the bidirectional relationship between character reading ability and orthographic awareness in Chinese, we tracked two separated samples of children in Beijing. The kindergarten sample, consisting of 96 children, was assessed three times in the first, second, and third years of kindergarten (K1, K2, K3) with 12-month intervals; the primary school sample, consisting of 204 children, was assessed four times in the first and second semesters of grade 1 (P1-S1; P1-S2), first semester of grade 2 (P2-S1) and grade 3 (P3-S1) with the first three waves at 6-month intervals and the final at 12-month intervals. The developmental patterns were analyzed in two cross-lagged models. Age and IQ were entered in the models as controlled variables, given their associations with the Chinese reading ability (e.g., Liu et al., 2013).

## Materials and Methods

### Participants and Procedures

Written and informed consent was obtained from the parents of all participants. All children in the two separated samples were native Mandarin speakers. None of them had hearing, visual, dyslexia, or other learning disabilities. All participants were tested individually in quiet rooms in their schools by trained psychology undergraduates/postgraduates. Chinese character recognition and orthographic judgment were measured in all testing occasions in both samples from K1 to P3. Radical knowledge was additionally measured in each of the four testing occasions in the primary school sample from P1 to P3. It took around 20–30 min to finish all the tasks.

There were 96 kindergarteners (49 boys) attending all the assessment points from K1 to K3. The mean age at the first assessment (2^nd^ semester of K1 in spring) was 49.8 months (*SD* = 6.6 months). The participants were recruited from two public (government) kindergartens. In Mainland China, formal literacy instruction has been prohibited at the kindergarten level since 1956, as to creating play-oriented learning environment, though unofficially literacy instruction may be conducted at home or private learning institutes (Li and Rao, 2000).

There were altogether 204 children from P1 to P3 (102 boys), recruited from one public primary school, which means that all students were from the same neighborhood. The number of participated students across the four testing times were 189, 203, 199, and 197 respectively. This small attrition was mainly due to students’ sick absence in school during the testing days. The mean age at the first assessment (1^st^ semester of P1 in fall) was 79.96 months (*SD* = 0.61 months). These participants were all recruited from the same public primary school.

### Measures

#### Chinese Character Reading

This task has been used successfully in the past to measure Chinese kindergartners’ character reading ability in Mainland China (Li et al., 2012). The list consists of 150 single-character characters, arranging in increasing difficulty level. The characters were selected from kindergarten and primary school textbooks (40 items from kindergarten and P1; 110 items from P2-6). The inclusion of all 150 items was to make the task sufficiently sensitive to capture children’s variation. The children were asked to read each character aloud; the task was terminated when a child failed to read 15 consecutive items. Each correct answer was credited one point.

#### Orthographic Awareness

In order to make the task to be age appropriate and sensitive, two sets of stimuli were used for the kindergarten and the primary school group respectively. We tested both positional awareness and semantic radical function in the primary school sample, but only positional awareness was assessed in the kindergarten sample, for mainly three reasons. First, formal literacy teaching was strictly forbidden in kindergarten in China, though there may be private tutorial at home or in education centers (Li and Rao, 2000). The analysis of primary school textbooks showed that 83% of required-to-learn characters are single-characters (e.g., 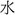
water) in the first semester of P1 (Shu et al., 2003). Many of these single-characters or their transformation [e.g., 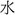 (water) – > 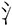
(radical meaning water)] are the radicals of compounding characters to be learned in higher grade levels. Second, previous evidence have shown that the development of positional knowledge is much earlier than the development of radical function knowledge, which typically appears in primary school ages (Chan and Nunes, 1998). Third, we pilot tested semantic radical function on eight kindergarten children, and floor effect was obtained. Kindergarteners have limited knowledge in semantic radical function. Thus, only positional awareness was formally assessed among the kindergarten sample.

For K1 to K3, the legitimateness of orthographic structure was assessed by 60 items including 30 real characters, 10 visual symbols, and 20 non-characters. The visual symbols were simple drawings, e.g., lines, circles, triangles, etc. Two types of non-characters were used, involving subtracting or adding strokes or reversing radical positions. The children were asked to identify whether each item was a real character. Each correct answer was credited one point with a maximum of 60 points. The same paradigm has been successfully used in Tong et al. (2009). Children at this age have mostly not developed the understanding of radical function (Qian et al., 2015), thus, radical function was not tested for this group of children. For P1–P3, two aspects of orthographic awareness, orthographic structure and semantic radical function, were included for a more comprehensive assessment, with the development of orthographic awareness in graders. Ninety items were used for assessing orthographic structure, consisting of 45 non-characters and 45 pseudo characters. The children were asked to answer whether the presented character was real or not. The inclusion of the 45 pseudo characters was designed only to balance yes/no answers and not counted in the final score calculation. Children would be more likely to respond “yes” for pseudo characters as they are orthographically regular. Using pseudo characters instead of real characters is to allow the whole task appear to be more challenging to primary school graders. Of the 45 non-characters, there were three types, with 15 items for each: (1) stroke error (unidentifiable stroke combination); (2) radical error (non-existent radical); (3) radical position error (switching radical positions). Each correct answer was credited one point with a maximum of 45 points for this orthographic structure task.

Formal instruction of reading and writing starts in P1 in Beijing and previous studies showed that children’s understanding of radical functional knowledge developed later than their positional knowledge (e.g., Ho et al., 2003a). Compared to phonetic radical function, evidence showed that children are more sensitive to semantic radical in primary school (Tong et al., 2017), maybe partly also due to that the phonetic cues are not reliable. Thus, from P1 to P3, children were additionally assessed by semantic radical knowledge task to tap the sensitivity to functional properties of radicals and the explicit knowledge of semantic radicals. A similar paradigm has been used successfully in a previous study (Tong and McBride-Chang, 2010). There were 40 items and each item in this task included a semantic radical (e.g., 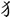) as the target and four options of pictures with different meanings. The children were asked to choose one from the four options representing the same meaning of the target semantic radical. Each correct selection was credited one point with a maximum of 40 points for the semantic radical function task.

Therefore, the maximum possible score of the orthographic awareness assessment for P1–P3 was 85.

### Data Analysis

Two cross-lagged models, one for the kindergarten and the other for the primary school sample were conducted with Mplus (Version 6.1) to depict the developmental changes of the relationship between character reading and orthographic awareness. The MLR estimator was used to estimate the parameters of the models. Missing data were replaced via the Full Information Maximum Likelihood (FIML) function.

Five indicators were adopted to evaluate the goodness-of-fit of the model: the chi-square test, Comparative Fit Index (CFI), Tucker–Lewis Index (TLI), Root Mean Square Error of Approximation (RMSEA), and Standardized Root Mean Square Residual (SRMR). The criteria of a good fit model suggested by previous researchers are: insignificant chi-square test, CFI and TLI that are equal to or greater than 0.95, SRMR that is equal or less than 0.08, and RMSEA that is equal to or less than 0.06 (Hu and Bentler, 1999).

## Results

Tables 1, 2 display the reliabilities, means, standard deviations and correlations of all tasks in the kindergarten and primary samples, respectively. The development of orthographic awareness and character reading progressed significantly across the years in both the kindergarten sample (K3 > K2 > K1 for both orthographic awareness and character reading) and the primary school sample (P3-S1 > P2-S1 > P1-S2 > P1-S1 for both orthographic awareness and character reading). Children performed on orthographic awareness significantly better than chance level at each time point in both the kindergarten (even at K1, *t* = 13.57, *p* < 0.001, significantly different from an accuracy at 30 items over a total of 60 items) and primary school samples.

**TABLE 1:** Reliabilities, means, SD and correlations of orthographic awareness and character reading in the kindergarten sample.

	***Max***	***Reliability***	***Means***	***SD***	**1**	**2**	**3**	**4**	**5**	**6**
(1) Orthographic awareness (K1)	60	0.665	36.85	4.98	_					
(2) Orthographic awareness (K2)	60	0.797	38.43	5.67	–0.080	_				
(3) Orthographic awareness (K3)	60	0.781	41.46	6.12	0.229^∗^	0.145	_			
(4) Character reading (K1)	150	0.979	3.74	11.03	0.174	0.282^∗∗^	0.410^∗∗^	_		
(5) Character reading (K2)	150	0.988	8.65	19.98	0.095	0.297^∗∗^	0.476^∗∗^	0.820^∗∗^	_	
(6) Character reading (K3)	150	0.990	14.94	24.08	0.179	0.263^∗∗^	0.531^∗∗^	0.749^∗∗^	0.906^∗∗^	_

*^∗^*p* < 0.05, ^∗∗^*p* < 0.01.*

**TABLE 2:** Reliabilities, means, SD and correlations of orthographic awareness and character reading in the primary school sample.

	***Max***	***Reliability***	***Means***	***SD***	**1**	**2**	**3**	**4**	**5**	**6**	**7**	**8**
(1) Orthographic awareness (P1-S1)	85	0.852	48.34	10.13	_							
(2) Orthographic awareness (P1-S2)	85	0.869	60.48	9.53	0.428^∗∗^	_						
(3) Orthographic awareness (P2-S1)	85	0.821	65.18	8.62	0.311^∗∗^	0.599^∗∗^	_					
(4) Orthographic awareness (P3-S1)	85	0.833	68.87	6.79	0.179^∗^	0.425^∗∗^	0.581^∗∗^	_				
(5) Character reading (P1-S1)	150	0.99	29.09	25.56	0.381^∗∗^	0.312^∗∗^	0.149^∗^	0.077	_			
6) Character reading (P1-S2)	150	0.99	54.36	27.22	0.472^∗∗^	0.360^∗∗^	0.217^∗∗^	0.096	0.776^∗∗^	_		
(7) Character reading (P2-S1)	150	0.99	70.36	23.46	0.469^∗∗^	0.402^∗∗^	0.246^∗∗^	0.135	0.727^∗∗^	922^∗∗^	_	
(8) Character reading (P3-S1)	150	0.99	98.87	16.04	0.414^∗∗^	0.358^∗∗^	0.293^∗∗^	0.155^∗^	0.541^∗∗^	754^∗∗^	0.822^∗∗^	_

*^∗^*p* < 0.05, ^∗∗^*p* < 0.01; the reliability of character reading was cited from a previous study (Li et al., 2012).*

The descriptive analysis of the subtasks of the orthographic awareness at each time point in the primary school sample was additionally calculated (available in Supplementary Analysis). Although the positional knowledge and semantic radical function were correlated at a relatively low level (approximately at 0.20 s), conceptually it is meaningful to have both positional knowledge and semantic radical function being taken into account in considering the general orthographic awareness.

**FIGURE 1:**
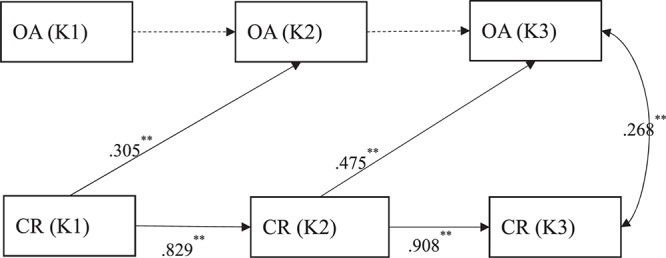
The relationship between orthographic awareness and character reading from K1 to K3. Standardized coefficients are reported. Dashed lines represent non-significant autoregressive paths of OA at different time points. Non-significant cross-lagged and correlation paths were not shown for the simplicity of presentation. OA = orthographic awareness, CR = character reading. ^∗^*p* < 0.05, ^∗∗^*p* < 0.01.

Cross-lagged models were conducted to test the relations between character reading and orthographic awareness. Orthographic awareness and character reading were set to be correlated at the same time point. In the kindergarten sample, the model fit were: χ^2^(4, *N* = 96) = 5.213, *p* = 0.2661, RMSEA = 0.056, CFI = 0.993, TLI = 0.976, SRMR = 0.040. As shown in Figure 1, the relationship between character reading and orthographic awareness was one-way only. Specifically, from K1 to K3, character reading predicted subsequent orthographic awareness (from K1 to K2, *B* = 0.157, *SE* = 0.047, β = 0.305, *p* < 0.01; from K2 to K3, *B* = 0.146, *SE* = 0.032, β = 0.475, *p* < 0.01). However, orthographic awareness did not predict subsequent character reading (both *p*s > 0.40).

**FIGURE 2:**
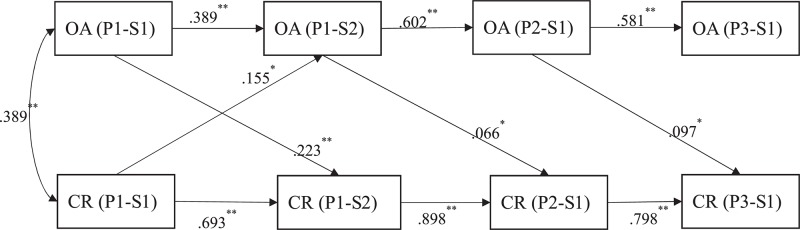
The relationship between orthographic awareness and character reading from P1 to P3. Standardized coefficients are reported. All non-significant paths were not shown for the simplicity of presentation. OA = orthographic awareness, CR = character reading. ^∗^*p* < 0.05, ^∗∗^*p* < 0.01.

In the primary school sample, the proposed cross-lagged model showed a good model fit, χ^2^(12, *N* = 204) = 11.964 (*p* = 0.45), RMSEA = 0.00, CFI = 1.00, TLI = 1.00, SRMR = 0.020. As shown in Figure 2, there was a bidirectional relationship between character reading and orthographic awareness between P1-S1 and P1-S2. The path from orthographic awareness to character reading was significant (*B* = 0.591, *SE* = 0.138; β = 0.223, *p* < 0.01), as well as the path from character reading to orthographic awareness (*B* = 0.057, *SE* = 0.025; β = 0.155, *p* = 0.019). In contrast, the relationship between character reading and orthographic awareness was observed to be one-way in the following years from P1-S2 to P3-S1. Specifically, during this one-and-a-half year period, orthographic awareness was observed to predict subsequent character reading (from P1-S2 to P2-S1: *B* = 0.163, *SE* = 0.074, β = 0.066, *p* = 0.024; from P2-S1 to P3-S1, *B* = 0.181, *SE* = 0.084, β = 0.097, *p* = 0.028). However, no significant prediction was found from reading to orthographic awareness in these later years (both *p*s > 0.70).

## Discussion

This study examined the 6-year developmental changes of the bidirectional relationship between character reading ability and orthographic awareness from K1 to P3. Cross-lagged analysis, with autoregressive effects addressed, was used to evaluate whether orthographic awareness enhanced gains in character reading ability and whether character reading ability supported gains in orthographic awareness. The results identified three developmental stages of the relationship over time. At the first stage, the longitudinal influence was originated from character reading to orthographic awareness, indicating a one-way pattern. In particular, character reading at K1 and K2 significantly predicted orthographic awareness at K2 and K3, respectively. Yet orthographic awareness at K1 and K2 did not predict subsequent character reading. In the second stage, orthographic awareness and character reading reciprocally predicted each other from the first semester of P1 to the second semester of P1. This bidirectional relationship diminished at the third stage and became a one-way influence from orthographic awareness to character reading from the second semester of P1 to the first semester of P3. No reverse contribution from character reading to orthographic awareness was found. Moreover, despite the lack of data in the higher primary grades, we anticipate this pattern may continue, until children reach a stable stage of character acquisition. Below, we discuss the possible mechanism underlying these developmental stages.

### Stage 1: The One-Way Contribution From Character Reading to Orthographic Awareness From K1 to K2 and K2 to K3

Orthographic awareness was detected by an orthographic judgment paradigm in kindergarten. Given the designed stimuli, this task mainly assessed the children’s awareness of the internal structure of characters and sensitivity of violation of positional constraint in Chinese. The stability of orthographic awareness from K1 to K3 was poor. Orthographic awareness at K1 did not predict orthographic awareness at K2, and orthographic awareness at K2 did not predict it at K3. We suspect that this instability of orthographic awareness performance may have been due to the limited orthographic knowledge the children had acquired at this stage. Formal teaching of writing is not encouraged – and is in fact forbidden – by the Education Bureau in preschools in Beijing (reported by Liang et al. (1997); cited in Li and Rao, 2000), to minimize early academic burden/competition, though literacy knowledge is incorporated in play-oriented activities. Even though the structure of Chinese orthography is quite complicated, without formal teaching children mostly acquire some orthographic knowledge via exposure to Chinese character (e.g., parent-child shared-book reading), implicit learning, or self-teaching.

The finding that reading performance at K1 and K2 predicted the gains of orthographic awareness at K2 and K3, respectively, is consistent with previous findings in alphabetic languages that repeated exposure to printed words facilitated the learning of orthographic knowledge (e.g., Barker et al., 1992; Kirby et al., 2008). The self-teaching theory (Share, 1995) may serve as a robust theoretical account to interpret the facilitation from reading to orthographic knowledge. The self-teaching hypothesis refers to the assumption that phonological decoding contributes to orthographic learning by creating the mapping between phonological representations onto orthographic representations. In other words, with the development of oral vocabulary and the repeated exposure to print, children gradually and autonomously acquire orthographic representations via decoding, and finally to ensure efficient word reading. Nation et al. (2007) summarized the particular process self-teaching: Children attempt to translate the novel printed words into its spoken equivalents; in return, each successful decoding provides an opportunity to acquire the word-specific orthographic information.

Chinese does not have such transparent phonology-orthography correspondence; and the orthographic units involved are radicals and characters in Chinese, instead of letters, letter clusters and words. Hence in Chinese, the nature of self-teaching is a bit different from its original hypothesis. Instead of establishing the arbitrary associations between a character and pronunciation, researchers have noticed that children can decode compounds into semantic radicals and phonetic radicals, and then apply the principles and knowledge of radicals to decode novel characters they encounter in reading (Luo et al., 2011). Through repeated exposure, children may discover the probabilistic patterns in the writing system and gradually construct orthographic representations. To conclude, reading Chinese characters provides an opportunity to sharpen sensitivity to orthographic structure and acquire the conventional knowledge of Chinese orthography. It is reasonable to infer that children with superior reading performances have had more reading experience in early kindergarten. In this study, the children with superior reading ability in K1 and K2 had superior orthographic awareness in K2 and K3, respectively.

Why was there no reversed effect from orthographic awareness to character reading at this stage? Explanations may be that children’s orthographic knowledge in K1 and K2 was inadequate or limited, and it takes time for orthographic knowledge to be developed more systematically to play an explicit role in learning Chinese. It is also possible that other cognitive-linguistic factors, such as phonological awareness (e.g., Siok and Fletcher, 2001), or sociocultural factors, such as parents’ attitudes and investment of time and money on children’s reading (e.g., Leseman and Jong, 1998; Chow et al., 2008) play more influential roles, especially in the early years.

### Stage 2: Bidirectional Relationship Between Orthographic Awareness and Character Reading From P1-S1 to P1-S2

The radical knowledge task in the present study, which directly assessed the understanding of the function of semantic radicals, was additionally used along with orthographic judgment task to capture children’s development of orthographic awareness during the primary school years, given the fact that children’s orthographic knowledge, particularly semantic aspects, develop rapidly in the early primary grades (e.g., Shu and Anderson, 1997; Ho et al., 2003a). Compared with Stage 1, a bidirectional relationship between character reading and orthographic awareness was demonstrated. With the start of formal instruction and the accumulated reading experiences with exposure to print generally, the longitudinal contribution from character reading to orthographic awareness continued to be evident during first 6 months of the primary school (P1-S1 to P1-S2).

Previous studies have provided empirical evidence that radicals serve as the primary functional units in processing compound characters (e.g., Luo et al., 2011), which explained why orthographic awareness, including radical knowledge, could predict children’s reading. Particularly, children with more radical knowledge may be better at inferring the meaning and the pronunciation of the encountered novel characters. As reported by Shu and Anderson (1997), children in primary school were able to identify semantic and phonetic radicals, and to use those radicals to derive the meaning or pronunciations of the character correctly.

It is evident that children can learn to read early before understanding orthographic knowledge. Chinese characters and words serve as input materials for self-teaching first. With an adequate input of characters and words, children start to acquire principles of orthographic structures. Then they attempt to make use of these principles to learn novel words. Thus, the reversed influence from orthographic awareness to reading should occur later than the influence from character reading to orthographic awareness. Additionally, a large proportion of the characters with which the children were in contact in preschool were simple ones, which cannot be decoded into radicals (Shu et al., 2003). Therefore, the acquirement of positional and functional principles of radicals in Chinese orthography may be very limited during preschool years. However, these simple radicals themselves may serve as radicals in compound characters in subsequent learning (Shu et al., 2003). The contribution of orthographic awareness to character reading, therefore, is expected to emerge after sufficient experience with characters, such as after formal literacy instruction, has taken place. This explains the bidirectional relations being observed during P1-S1 to P1-S2.

### Stage 3: One-Way Influence From Orthographic Awareness to Subsequent Character Reading From P1-S2 Toward P3-S1

With the learning progressing from P1 to P2 and P3, orthographic awareness continuously predicted the development of character reading; however, the reversed contribution from character reading to orthographic awareness disappeared. Although the characters encountered in higher grades are more visually complicated (e.g., more strokes or radicals), the required orthographic knowledge is not getting more difficult and the basic structures and rules of Chinese orthography largely remain the same. In fact, the characters learned in higher grades are more regular rather than irregular (Shu et al., 2003). In the current data, the increasing rate of Children’s orthographic awareness, especially semantic radical knowledge, was most rapid during P1, and slowed down in P2 and P3. It is possible that, in approaching this third stage, the learning of orthographic knowledge by reading inputs is somehow saturated. Stimulation by reading can no more promote the development of orthographic awareness. Alternatively, other stimulations, such as writing, may push further the development of orthographic knowledge in middle and high primary grades, given that writing may serve as a platform to deeply integrate orthographic, phonological, and semantic components as found in learning Chinese (Tan et al., 2005) and English (Ouellette, 2010), as well as for Chinese children learning English as a second language (Lin et al., 2016).

The majority of the characters are regular compounding (Shu et al., 2003), despite the slow increase of knowledge scope, the skill of observing and analyzing newly encountered characters may be more proficient with reading experiences (Shu et al., 2003), which lead to the consistent contribution from orthographic awareness to character reading.

It is worth noting that the association between orthographic awareness and reading may differ across scripts. Our finding that orthographic plays a salient role in predicting reading since the entrance of primary school till P3 (refer to Stages 2 and 3) are in contrast to some recent findings in English (e.g., Burt, 2006; Deacon et al., 2012; Conrad and Deacon, 2016), which reported a one-way contribution from word reading to orthographic skills in primary school children. Deacon et al. (2012) found that word reading predicted orthographic skills from P1 to P3 with vocabulary, non-verbal reasoning, and phonological awareness controlling, but no significant prediction in the reverse direction. This discrepancy is likely caused by the distinctive features of Chinese and English orthographies. Burt (2016) claimed that an orthographic processing skill is not a dependent one that is distinct from phonological processing. Given the fact that orthographic processing skill in English mainly involves letter and letter cluster identification, which automatically activate phonological processing, this claim makes much sense. However, in Chinese, the situation is quite different. As described in the introduction, Chinese orthography have several levels of structure (stroke, stroke pattern, radical, character). Stroke, stroke patterns, and some radicals are unpronounceable (e.g., semantic radicals such as 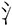, 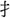). Therefore, the visual-orthographic processing in Chinese is relatively independent from phonological processing, which further supports an important role of orthographic awareness in Chinese reading.

## Conclusion

One limitation of the current study is the measure of orthographic awareness, particularly the lack of assessing phonetic radical knowledge. We chose to test the semantic radical knowledge in our lower-grades sample, given the evidence that semantic radical function played a more important role in Chinese reading, as compared to phonetic radical in primary school (Tong et al., 2017). Despite the lack of phonetic radical, the impact on the overall developmental pattern of reading and orthographic awareness might be minor, due to a relatively less important role of phonetic radical function in reading. This warrants further empirical investigation. Another limitation may be that the study involved two separate samples with both cross-sectional and longitudinal designs. Although this introduces clear merits, such as less bias from historical effects (e.g., curriculum changes) and saving project span, it would be very interesting to track the same samples longitudinally across the same grade levels. Additionally, the study lacked the control of other cognitive and metalinguistic skills, such as working memory and morphological awareness. However, we also believe that the autoregression serves the strongest control in predicting longitudinal developmental outcomes in the cross-lagged analysis. Further studies should include other influential cognitive and metalinguistic skills to depict a broader developmental picture of Chinese reading acquisition in young children. Finally, the study did not distinguish orthographic skills in the lexical and sub-lexical sub-components (Commissaire and Besse, 2019). It would be interesting to consider the developmental picture of these sub-components in relation to word reading across scripts.

Despite the limitations, the findings of the present study depict the developmental picture of the changed relationship between Chinese character reading and orthographic awareness over time. In the 6-year developmental course from the entire kindergarten period to the first 3 years of primary schooling in Beijing children, three developmental stages were identified. There are great contextual differences across different Chinese societies (e.g., Hong Kong, Taiwan), and these factors may influence the onset, duration of each stage, and strength of the associations between orthographic awareness and Chinese character reading development. These interesting variations warrant further investigations. The beginning readers in this study demonstrated impressive abilities for discovering or extracting orthographic regularities with increased reading ability. Additionally, in contrast to some findings in English (Burt, 2006), this study has demonstrated a sustainable influence of orthographic awareness in reading development uniquely in Chinese.

## Data Availability Statement

All datasets generated for this study are included in the manuscript/Supplementary Files.

## Ethics Statement

This study was carried out in accordance with the recommendations of “Beijing Normal University and The Education University of Hong Kong Ethics Review Committee” with written informed consent from all subjects. All subjects gave written informed consent in accordance with the institutional requirements. The protocol was approved by the “Beijing Normal University and The Education University of Hong Kong Ethics Review Committee.”

## Author Contributions

DL was responsible for conceptualization, and writing and approving the manuscript. DL and HL were data owners and responsible for approving the final draft. JM supported on literature review and drafting. YL supported on data analysis.

## Conflict of Interest

The authors declare that the research was conducted in the absence of any commercial or financial relationships that could be construed as a potential conflict of interest.
