# Supplementary material for: Developmental Changes in the Relationship Between Character Reading Ability and Orthographic Awareness in Chinese
Source: Front Psychol. 2019 Oct 25;10:2397. doi: 10.3389/fpsyg.2019.02397 (PMC6824357; doi:10.3389/fpsyg.2019.02397)
Supplement: Supplementary file 1 [file Table_1.DOCX]

*Supplemental analysis*

*Means and SD of Orthographic Awareness Subtasks at Each Time Point in the Primary School Sample*

|  | Max | *Means* | *SD* | *Means* | *SD* | *Means* | *SD* | *Means* | *SD* |
| --- | --- | --- | --- | --- | --- | --- | --- | --- | --- |
| Subtasks |  | *P1-S1* | | *P1-S2* | | *P2-S1* | | *P3-S1* | |
| 1. OA_stroke error | 15 | 13.79 | 2.64 | 14.36 | 1.75 | 14.55 | 1.31 | 14.76 | 1.04 |
| 2. OA_radical error | 15 | 6.97 | 3.70 | 9.03 | 3.59 | 9.58 | 3.43 | 10.25 | 3.22 |
| 3. OA_radical position error | 15 | 9.72 | 4.09 | 12.23 | 3.38 | 13.36 | 2.79 | 14.11 | 2.01 |
| 4. OA_structure (1+2+3) | 45 | 30.49 | 8.61 | 35.63 | 7.12 | 37.49 | 6.24 | 39.12 | 5.06 |
| 5. OA_semantic radical function | 40 | 17.69 | 4.65 | 24.85 | 4.81 | 27.68 | 4.42 | 29.75 | 4.53 |
| 6. OA_all | 85 | 48.34 | 10.13 | 60.48 | 9.53 | 65.18 | 8.62 | 68.87 | 6.79 |
